# Deathtouch: The Long and Selective Reach of Proneurotrophin Shapes Neurodegeneration after Concussive Brain Injury

**DOI:** 10.1523/ENEURO.0340-23.2023

**Published:** 2023-10-19

**Authors:** Chia-Wei Yeh, Andrew Huang

**Affiliations:** 1 Department of Molecular, Cell and Systems Biology, University of California, Riverside, Riverside, California 92521; 2 Neuroscience Graduate Program, University of California, Riverside, Riverside, California 92521; 3 Biomedical Sciences Graduate Program, University of California, Riverside, Riverside, California 92521

Traumatic brain injury (TBI) affects millions of people annually and is a leading cause of long-term disability. Individuals with this condition commonly manifest behavioral, psychiatric, and cognitive dysfunctions, yet there is inadequate understanding of underlying mechanisms, posing challenges for effective treatments. TBI often arises from physical trauma to the head resulting in focal or diffuse injury, long-term cell loss, and tissue damage depending on injury type. Focal injuries can be modeled with the controlled cortical impact (CCI), while fluid percussion injury (FPI) models diffuse concussive injuries. Furthermore, each type of injury can be further classified based on severity (e.g., mild, moderate, and severe), each with their own unique pathophysiology. In mild TBI, diffuse injury leads to cell loss, diffuse axonal injury, and related circuit alterations in both afferent and efferent networks connecting to the injured cortical area (Krishna et al., 2020). It is recognized that dysfunction of distributed networks after brain injury could contribute to behavioral, emotional, and cognitive postconcussive symptoms. Several neuromodulatory systems participating in the cognitive function, such as the cholinergic, noradrenergic, and dopaminergic systems, are known to be affected by TBI (Shin and Dixon, 2015). However, whether specific subcortical networks are altered after injury and the underlying molecular mechanisms is not known. The study by Dasgupta et al. (2023) addresses this important issue by examining the effect of mild to moderate concussive TBI on basal forebrain (BF) cholinergic neurons (CNs) and locus coeruleus (LC) noradrenergic neurons, both of which are key players in cognitive function, including learning, memory, arousal, and attention.

Dasgupta et al. (2023) build on their earlier study examining mechanisms of local cell death in the CCI model (Montroull et al., 2020) in which they demonstrated a role of neurotrophin (NT) receptor p75NTR in the cell death at injury site. They identified that cortical neurons near the injured site contained higher levels of p75NTR and showed evidence of apoptosis. Importantly, attenuating NT-p75NTR signaling using either siRNA or neutralizing antibodies targeting NTs abolished the CCI-induced apoptosis at the injury site. These findings revealed a critical role of NTs and p75NTR in the regulation of cell death at the impact site in focal TBI. Interestingly, apart from apoptosis, NT signaling also participates in the development, maturation, survival, and programmed cell death in both the BF and LC (Friedman et al., 1993; Boskovic et al., 2019). While it appears paradoxical that p75NTR-mediated signaling can support both cell survival and cell death, this is a consequence of the binding between different NT receptor subtypes and immature/mature NTs. The mature NTs and the uncleaved NT precursors, proNTs, activate distinct signaling pathways via binding onto diverse NT receptors including p75NTR, tyrosine receptor kinase (Trk) family, and sortilin. The diverse combination between the NTs/proNTs and their receptors results in multiple and complex cellular functions. Here, Dasgupta et al. (2023) examined whether diffuse injury leads to increases in proNT in the underlying injured cortex and whether this could impact the survival of p75NTR-expressing neurons in the BF and LC.

Using the mild to moderate concussive FPI model of TBI, Dasgupta et al. (2023) investigated the effect of TBI on the afferent inputs to the injured cortex and the underlying mechanisms of FPI-induced afferent loss. They first demonstrated an increased expression of proNTs selectively at the cortex underlying the injury site after FPI, but not in the BF and LC. Although proNT levels were not elevated in the BF, the number of BF CNs expressing p75NTR was significantly reduced, indicating cell death. This only occurred on the side of injury, suggesting that cortical injury could retrogradely promote degeneration of afferent BF neurons. Interestingly, mice lacking p75NTR did not show injury-induced cell loss in the BF despite having increases in proNT at the injury site, identifying that proNT-p75NTR signaling as critical for FPI-induced BF neuronal loss. Since the level of proNTs at the BF was not altered after FPI, it stands to reason that p75NTR could only have been activated by elevated proNTs at the cortical axonal terminal of BF CNs. To specifically test the role for retrograde axonal transport of proNT in the BF CN, they adopted a microfluidic culture system where the extracellular environments of the axonal compartment could be isolated from the soma and stimulated independently. Consistent with the *in vivo* studies, they demonstrated that either knocking out p75NTR or blockade of dynein-mediated retrograde transport prevented the axonal proNT-induced apoptosis, indicating that axonal proNTs promoted cell loss in a p75NTR-dependent and retrograde transportation-dependent manner. Compared with the BF inputs, retrograde tracing showed that p75NTR-expressing LC neurons did not directly target the injured cortex. Consistently, although tyrosine hydroxylase-positive LC noradrenergic neurons expressed p75NTR, they show limited post-traumatic degeneration. These data suggest that retrograde signaling is necessary for neuronal degeneration in injured cortex-projecting BF CNs after concussive brain injury.

Overall, these findings demonstrate that diffuse TBI promotes death of BF CNs, which projects to the injured cortex, mediated by retrograde proNT-p75NTR signaling. These findings suggest that NT-p75NTR-mediated cell death could underlie TBI-induced long-term cognitive disorders. Degeneration of BF CNs after TBI could contribute to the post-traumatic increase in risk for neurodegenerative disorders such as Alzheimer’s disease. In this regard, the authors could have included functional assays to determine whether reduced post-traumatic BF CN loss in mice lacking p75NTR improved long-term cognitive outcomes. The study raises several interesting questions and future directions: (1) what are the proteins or secondary messengers transported retrogradely by dynein that contribute to “the deathtouch”? (2) Are there ways to therapeutically target BF CNs to make them resistant to proNT-p75NTR signaling-mediated cell death? Understanding the underlying mechanisms of the TBI-induced dysfunction of distributed networks would be an important step toward developing therapeutic strategies to limit secondary neurologic disorders and cognitive impairments after TBI.

## Synthesis

Reviewing Editor: Viji Santhakumar, University of California Riverside

## References

[B1] Boskovic Z, Meier S, Wang Y, Milne MR, Onraet T, Tedoldi A, Coulson EJ (2019) Regulation of cholinergic basal forebrain development, connectivity, and function by neurotrophin receptors. Neuronal Signal 3:NS20180066. 10.1042/NS20180066 32269831 PMC7104233

[B2] Dasgupta S, Montroull LE, Pandya MA, Zanin JP, Wang W, Wu Z, Friedman WJ (2023) Cortical brain injury causes retrograde degeneration of afferent basal forebrain cholinergic neurons via the p75NTR. eNeuro 10:ENEURO.0067-23.2023. 10.1523/ENEURO.0067-23.2023 PMC10467018 37558465

[B6] Friedman WJ, Ibáñez CF, Hallböök F, Persson H, Cain LD, Dreyfus CF, Black IB (1993) Differential actions of neurotrophins in the locus coeruleus and basal forebrain. Exp Neurol 119:72–78. 8432352 10.1006/exnr.1993.1007

[B3] Krishna G, Beitchman JA, Bromberg CE, Currier Thomas T (2020) Approaches to monitor circuit disruption after traumatic brain injury: frontiers in preclinical research. Int J Mol Sci 21:588. 10.3390/ijms21020588 31963314 PMC7014469

[B4] Montroull LE, Rothbard DE, Kanal HD, D'Mello V, Dodson V, Troy CM, Zanin JP, Levison SW, Friedman WJ (2020) Proneurotrophins induce apoptotic neuronal death after controlled cortical impact injury in adult mice. ASN Neuro 12:1759091420930865. 10.1177/1759091420930865 32493127 PMC7273561

[B5] Shin SS, Dixon CE (2015) Alterations in cholinergic pathways and therapeutic strategies targeting cholinergic system after traumatic brain injury. J Neurotrauma 32:1429–1440. 10.1089/neu.2014.3445 25646580 PMC4842943

